# A South African Indian population group dataset for breast cancer and *BRCA1/2* variants

**DOI:** 10.1016/j.dib.2022.108180

**Published:** 2022-04-14

**Authors:** H.M.V.E. Combrink, N.C. van der Merwe, R. Katarya, K. de Wet, M.H. Motloung

**Affiliations:** a Interdisciplinary Centre for Digital Futures, University of the Free State, South Africa; b Office of the Dean, Economic and Management Sciences, University of the Free State, South Africa; c Division of Human Genetics, National Health Laboratory Service, Universitas Hospital, Bloemfontein, South Africa; d Division of Human Genetics, Faculty of Health Sciences, University of the Free State, Bloemfontein, South Africa; e Department of Computer Science and Engineering, Delhi Technological University, Shahbad Daulatpur, Delhi, India; f Department of Sociology, University of the Free State, South Africa

**Keywords:** Ethnic group, *BRCA1/2*, VUS, Polymorphisms, Cancer type, Breast cancer, Ovarian cancer, Cancer stage, Pathogenic mutation status

## Abstract

No datasets are available on the basis of confirmed familial breast cancer patients and their respective mutation status for the South African Indian population. This dataset contains information collected in South Africa specifically for the South African Indian population (n = 223). The data involved a combined collection of laboratory confirmed genetic testing results, accompanied by immunohistochemical and anatomical pathology results. Various patient self-identified variables were also in measured. All the immunohistochemistry and anatomical pathology results were confirmed by a trained medical pathologist. Furthermore, mutation status and the detection of either benign polymorphisms or variants with an unknown clinical significance at the time of data collection were also reported. This dataset contains variables in a tabular format which includes the accompanying variables for each patient. All the patient-specific data were deidentified which allows for further exploration of the data with respect to the demography, immunohistochemistry and genetic material. This dataset has the potential to serve as the backbone for translational research and inquiries into this unique South African population.

## Specifications Table

**Table:** 

Subject	Health and medical sciences: Clinical Genetics
Specific subject area	The dataset contains tabular patient data with *BRCA1/2* genetic information specific for high-risk South African Indian patients
Type of data	Patient-based clinical and *BRCA1/2* genetic variant data
How the data were acquired	The data in this study was extracted from a SQL database stored within the Human Genetics department at the University of the Free State. The SQL database is linked to a series of front-end forms that allows the medical scientists in the laboratory to capture research-based information once ethical clearance has been obtained for a specified study. The dataset was attained by applying for formal ethical clearance through the correct ethics committee (University of the Free State (ECUFS 107/2014) and the Department of Health, KwaZulu-Natal (HRKM194/14). Once obtained, the hospital manager, and the laboratory manager provided further permission for the use of the data. Finally, the lead laboratory scientist in charge of the system responsible for the capturing of this data gave the final approval. Once extracted, the specifications of the ethical approval were followed in terms of the type of data shared and extracted from the database. This included the deidentification of the patient's information and the removal of any sensitive information that may be linked to the patient.
Data format	Raw tabular data
Description of data collection	Patients diagnosed with breast and/or ovarian cancer is referred to a specialist physician, to which the specialist physician completes a request form (Figure 1). If a biopsy was performed on the cancer or tissue type at the time of the consultation, then this information is also captured on the patient request form. The patient-based data involving patient demographics and the supporting clinical information was captured from a formal pathology request form (Figure 1). All the genetic information was captured in an online database after verification by two independent senior medical scientists and the requesting physician. The genetic information was identified by conducting a series of laboratory genetic tests on the DNA extracted for each of the patients in the cohort, and the results captured and validated by medical scientists. Sanger sequencing was then performed on the specified genetic region identified with an anomaly to confirm the specific genetic diagnosis, and this final confirmation was also captured on a secure database. All the information was then validated by a medical scientist in a secure database to ensure that the supporting demographic and clinical data corresponds with the correct patient in the database and is linked to the correct genetic information identified.
Data source location	National Health Laboratory Services, University of the Free State Bloemfontein, Free State South Africa Latitude and longitude -29.1075973/26.1924506:
Data accessibility	Repository name: A South African Indian Population Group Dataset for Breast Cancer and BRCA1/2 Variants Data identification number: DOI:10.17632/7z5fxz6r6x.4 Direct URL to data: https://data.mendeley.com/datasets/7z5fxz6r6x/4
Related research article	Combrink, H. M., Oosthuizen, J., Visser, B., Chabilal, N., Buccimazza, I., Foulkes, W. D., & van der Merwe, N. C, Mutations in BRCA-related breast and ovarian cancer in the South African Indian population: a descriptive study, Cancer Genetics. 259-259 (2021) 1–6. https://doi.org/10.1016/j.cancergen.2021.06.002

## Value of the Data

• All the data in the dataset was verified by a clinician and two independent senior medical scientists and underwent de-identification according to appropriate guidelines after ethical clearance to verify the quality and integrity of the data
• Currently, no other dataset is available providing characteristics for the South African Indian population with regard to familial breast cancer and the associated clinical characteristics
• The dataset contains de-identified information about the immunohistochemistry results associated with specific genetic variants, as well as unique clinical features
• The dataset in this context is useful for eHealth classification models due to a lack of freely accessible datasets for the South African Indian Population
• Not all the variables in association with this dataset have been explored and as a result, the dataset provides the opportunity for an exploratory data analysis
• The dataset involves a unique admixed population, which differs with regards to their *BRCA1/2* mutation spectrum from mainland India

## 1 Data Description

**Fig. 1:**
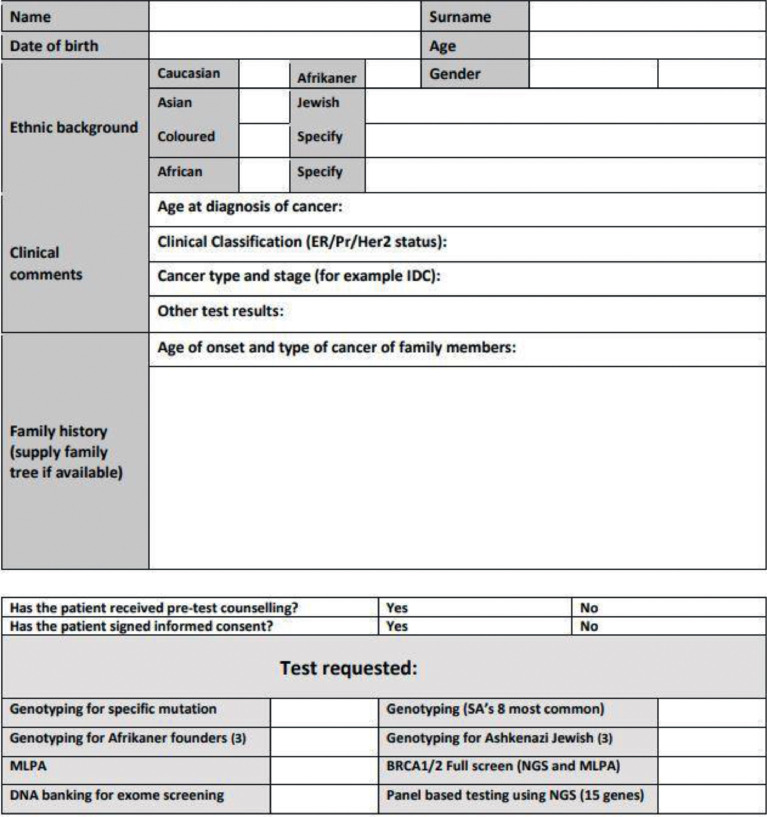
Patient request form.

The raw data file within the final dataset consolidates all the information presented in Table 1, Table 2, Table 3 for each of the 223 patients in this study. The data in the final dataset is presented in one of two formats, as tabular data in a .csv format or as a JSON format in a .txt file. The patient request form is the first file within the dataset. The demographic and clinical data was determined through consultation with a specialised medical physician (Fig. 1).

**Table 1:** Demographic data variables.

Name of variable	Data type	Description
d_age_distribution	Categorical	One of three age distributions of the patients. The ages of the patient were aggregated to represent only one of three stratified age groups, as part of the selection criteria of risks associated with the scope of the original research (please refer to Combrink et al.*,*[1] for further information)
d_biological_gender	Categorical	One of two possible options for biological gender
d_ethnic	Categorical	Only one specified ethnic group
d_cancer_in_family	Categorical	Different cancers present within the known family. Accompanied with each of the request forms were the other cancer types within a given pedigree and related family. These cancer types were recorded in this variable
d_affected_family_members	Categorical	Four possible categories of affected family members. This variable indicates whether or not there were other members within the related family of the index patient affected with cancer. Within this variable, the number of affected family members were also shared as per the scope of the original research (please refer to Combrink et al.*,*[1] for further information).

**Table 2:** Clinical data.

Name of variable	Data type	Description
c_mutation_risk	Categorical	What the clinical risk is of a patient to carry a pathogenic variant
c_affected_un	Categorical	Whether or not the patient is affected with breast cancer
c_affected_cancer_type	Categorical	What specific cancer the patient is affected with
c_er_marker	Categorical	Was the cancer ER positive or negative? ER refers to whether the cancer grows in the presence of the hormone estrogen
c_pr_marker	Categorical	Was the cancer PR positive or negative? PR refers to whether the cancer grows in the presence of the hormone progesterone
c_her2_marker	Categorical	Was the cancer HER2 positive or negative? HER2 refers to a protein called the human epidermal growth factor receptor 2 which promotes the growth of cancer cells

**Table 3:** Genetic data.

Name of variable	Data type	Description
g_type_of_variant detected	Categorical	The type of variant detected in patient
g_variant_mutation	Categorical	Whether the detected variant was pathogenic
g_variant_vus	Categorical	Whether the detected variant had an unknown clinical significance (VUS)
g_variant_benign	Categorical	Was the detected variant benign
g_gene	Categorical	Which one of the two genes was the variant detected in
g_genetic_result	Categorical	The name of the genetic variant according to its nomenclature. This variable provides the scientific annotation of the specific genetic change (variant) relative to the position within the *BRCA1* and *BRCA2* genes.
g_deletion	Categorical	Did the variant cause a deletion in the gene
g_terminate_gene	Categorical	Whether or not the variant caused a termination in the formation of the protein
g_frameshift	Categorical	Whether or not the variant caused a translational modification in the polypeptide chain
g_t>a	Categorical	Was the genetic variant a pyrimidine to purine change
g_t>c	Categorical	Was the genetic variant a pyrimidine to pyrimidine change
g_t>g	Categorical	Was the genetic variant a pyrimidine to purine change
g_a>t	Categorical	Was the genetic variant a purine to pyrimidine change
g_a>g	Categorical	Was the genetic variant a purine to purine change
g_a>c	Categorical	Was the genetic variant a purine to pyrimidine change
g_g>c	Categorical	Was the genetic variant a purine to pyrimidine change
g_g>t	Categorical	Was the genetic variant a purine to pyrimidine change
g_g>a	Categorical	Was the genetic variant a purine to purine change
g_c>g	Categorical	Was the genetic variant a of pyrimidine to purine change
g_c>a	Categorical	Was the genetic variant a of pyrimidine to purine change
g_c>t	Categorical	Was the genetic variant a pyrimidine to pyrimidine change

In the final dataset, the patient name, surname, date of birth and age were removed and only the age range was kept. Furthermore, in the final dataset, only patients classified from the South African Indian population were used. The data was stratified into three overarching components namely: demographic insights, clinical evidence and genetic information. The final dataset (which includes the genetic screen and the data from the patient request form) is presented in a raw tabular data format, and consist of demographics data (prefix d), clinical- (prefix c) and genetic variables (prefix g), all of which are aimed to provide a clear assessment of what is needed to make an informed diagnosis for familial breast cancer in this population group. All of the variables within the dataset were categorical, and the validity of each variable was confirmed by either a medical officer, medical specialist or medical scientist. The demographic data is what was identified at time of first consultation (Table 1). Apart from the description of the data presented in Table 1, the variable “d_cancer_in_family” has different classifications underneath it which includes all the reported cancer types within the genetic and related family members of the index patient. The index patient is the patient that underwent genetic screening.

The clinical data is a combination of family-specific clinical questions aimed at determining disease risk, in combination with confirmed immunohistochemistry and anatomical pathology results for each patient (Table 2). For some of the patients this data was not available as the genetic screening and immunohistochemistry and anatomical pathology testing occurred simultaneously. As a result, for these patients not all the clinical data was captured. All of the patients in this dataset had a confirmed cancer diagnosis and the primary cancer type (breast cancer or ovarian cancer) was confirmed by a trained medical specialist prior to the consultation with the patient. Also included is the associated risk of each patient in the dataset according to the breast cancer genetic screening guidelines of South Africa (“c_mutation_risk”).

The genetic information is based on the laboratory-specific test results for each of the patients (Table 3). All of the data collected was verified and captured by trained clinicians and medical scientists. Only anomalies within the genetic screening results were reported, as there are ample reference genetic sequences available for normal *BRCA1/2* genes [2]. The *BRCA1/2* genetic variants were annotated according to specified international nomenclature using the NCBI chromosomes and transcript reference sequences for each of the genes (NC_000017.11, NM_007294.4 and NC_000013.11, NM_000059.3). The genetic data in the dataset is unique to the South African Indian population. Within the dataset, the type of genetic result, if the genetic result caused a frameshift mutation, deletion, or a premature termination was included. There are genetic variations that were classified as variants with an unknown clinical significance (VUS). These variants did not at the time of publication have a known clinical significance and these variants are still under investigation. In addition to this, if the genetic change was as a result of a shift from either a purine to a pyrimidine (or vice versa), or no change – then it was reported for each nucleotide combination.

The categorical parameters in this dataset contains a variety of different variables related to clinical genetic information specific to the South African Indian population.

## 2 Experimental Design, Materials and Methods

**Fig. 2:**
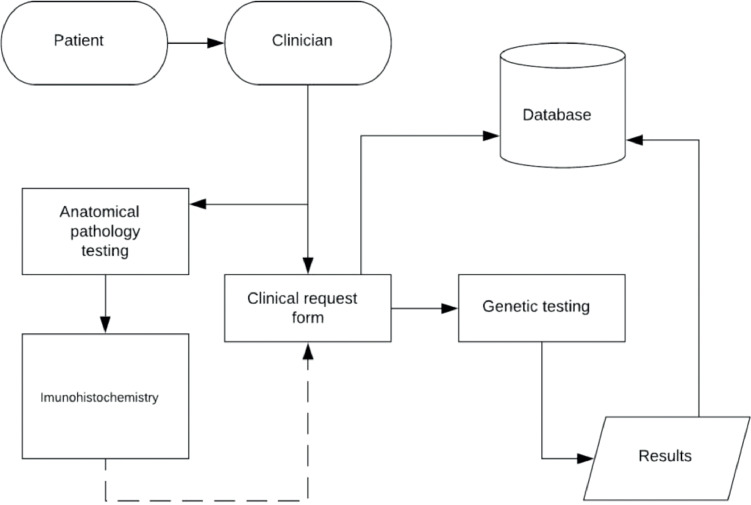
Data collection cycle.

The study conducted followed a descriptive experimental design whereby data was directly captured and recorded from patient request forms and laboratory confirmed genetic screening and sequencing results. The entire cascade of events that led up to genetic testing commences with a patient consulting a clinician and the data captured on a request form. Upon diagnosis, the treating clinician typically refers samples for pathological confirmation of disease before transferring the patient to an oncologist, this generates the clinical features data within the dataset. If the supporting clinical criteria are met, then the risk data are generated based on the patient's age of onset and/or family history. Based on the risk that the patient is likely to carry a disease-causing *BRCA1/2* variant, the patient is referred for genetic testing. Genetic testing generates the data related to the genetic features in the dataset. This entails the extraction and purification of DNA from blood representative of a specific patient. Once extracted, analysis is conducted using a series of investigative laboratory procedures, to collectively detect all the various types of potential variants, such as single nucleotide changes or copy number variants. All tests are founded on the exponential multiplication of highly specific areas in each of these genes to identify changes on both DNA and protein level. If any anomaly is detected (that is, a genetic result that is different from the norm), it will be scrutinized using various methodologies, including DNA sequencing, to confirm its presence. Once the presence of an anomaly is detected, the information is captured. The genetic screening process is broken up into several stages due to the large size of *BRCA1* and *BRCA2* (more than 80 different regions experimentally screened per sample) (Fig. 2).

There are instances where all processes occurred simultaneously (anatomical pathology testing, immunohistochemistry, and genetic testing). Under such circumstances, there will be no supporting data from the pathology results captured on the patient request form. In such an event where the first two tests were conducted prior to the request for genetic testing, the clinical information will accompany the clinical request form, where after genetic testing will commence. Once a pathogenic anomaly is confirmed, the information is stored in a secure database. This database contains all the relevant information about the patients that came from the patient request form as well as all the genetic data that was generated and confirmed through Sanger sequencing. Some of the variables and variable fields undergo feature engineering steps. These steps are introduced to deidentify the samples and stratify the information according to the experiments conducted. Each of the patients in the study underwent a further clinical justification step according to their risk profile which was further stratified into low, moderate, and high risk. At every step of the process, all the information was signed off by a senior medical scientist or medical specialist. The data is also collected and validated at each step of the data collection cycle by two medical scientists (Fig. 2).

## Ethics Statements

Ethical approval for the study was obtained from the Health Sciences Research Ethics Committee at the University of the Free State (ECUFS 107/2014) and the Department of Health, KwaZulu-Natal (HRKM194/14). All the patients in this study completed an informed consent form. Only patients that allowed for further analysis of their samples were included in the dataset. Within this study and with this dataset there were no (none) patients that objected to the storage, study and follow-up analysis using their information. All of the procedures and standards of this study followed the strict and clear protocol of the project plan, under the guidance of the ethical measurements used in this study.

By adhering to all the ethical guidelines, we confirm that informed consent was obtained for all participants in this study. The research in this study was carried out in accordance with the code of ethics from the South African Medical Research Council, the University of the Free State Medical School Ethics committee, and the Department of Health, KwaZulu-Natal which are in accordance with the World Medical Association's Declaration of Helsinki.

## CRediT authorship contribution statement

**H.M.V.E. Combrink:** Conceptualization, Methodology, Formal analysis, Writing – original draft. **N.C. van der Merwe:** Resources, Conceptualization, Validation, Data curation, Writing – review & editing. **R. Katarya:** Conceptualization, Methodology. **K. de Wet:** Writing – review & editing, Writing – original draft. **M.H. Motloung:** Writing – review & editing, Writing – original draft.

## Declaration of Competing Interest

The authors declare that they have no known competing financial interests or personal relationships that could have appeared to influence the work reported in this paper.

The authors declare the following financial interests/personal relationships which may be considered as potential competing interests:

The authors declare that they have no known competing financial interests or personal relationships that could appear to influence the work reported in this paper.

## Data Availability

A South African Indian Population Group Dataset for Breast Cancer and BRCA1/2 Variants (Original data) (Mendeley Data).
